# CCL17/CCR4 Axis Promotes Hematoma Clearance via ERK/AP1/SRA‐Mediated Microglial Polarization After Intracerebral Hemorrhage

**DOI:** 10.1111/cns.70288

**Published:** 2025-02-25

**Authors:** Xianglan Chen, Yao Wang, Junjie Jin, Peng Jin, Shuixiang Deng

**Affiliations:** ^1^ Department of Critical Care Medicine HuaShan Hospital, Fudan University Shanghai China; ^2^ Department of Intensive Care Medicine, the First Affiliated Hospital of USTC, Division of Life Sciences and Medicine University of Science and Technology of China Hefei Anhui China

**Keywords:** CCL17/CCR4, ERK/AP1/SRA pathway, hematoma clearance, intracerebral hemorrhage, microglial polarization

## Abstract

**Background:**

Our previous studies demonstrated that CCL17 and its receptor CCR4 play crucial roles in neuroinflammation and microglial activation following intracerebral hemorrhage (ICH). However, the specific mechanisms by which the CCL17/CCR4 axis regulates microglial polarization and hematoma clearance remain unclear.

**Aims:**

This study investigates how the CCL17/CCR4 signaling pathway modulates microglial phenotype transition and enhances hematoma resolution after ICH, building upon our earlier findings showing CCR4's involvement in neuroinflammatory responses.

**Methods:**

Using CRISPR‐mediated CCR4 disruption and CCR4 overexpression approaches in a mouse ICH model, we examined neurological outcomes, inflammatory responses, and hematoma volumes. We further evaluated the therapeutic potential of recombinant CCL17 administration. The downstream ERK signaling pathway's role in CCL17/CCR4‐mediated microglial function was investigated through pharmacological inhibition.

**Results:**

CCR4 knockout exacerbated neurological deficits, increased neuroinflammation, and enlarged hematomas. In contrast, enhancing CCR4 expression or administering recombinant CCL17 improved functional recovery and provided neuroprotection. Mechanistically, CCL17/CCR4 signaling activated the ERK/AP1/SRA pathway, promoting anti‐inflammatory, phagocytic microglial polarization, evidenced by increased CD206 and SRA expression. ERK inhibition reversed these protective effects.

**Conclusion:**

Our findings extend previous work by revealing that the CCL17/CCR4 axis enhances hematoma clearance through the ERK/AP1/SRA pathway‐mediated microglial polarization. This mechanism represents a promising therapeutic target for ICH treatment.

## Graphical abstract

The study identifies the critical role of the CCL17/CCR4 axis in microglial polarization and hematoma clearance following intracerebral hemorrhage. By activating the ERK/AP1/SRA signaling pathway, CCL17/CCR4 enhances neuroprotection and functional recovery, providing a potential therapeutic target for improving outcomes in stroke patients.

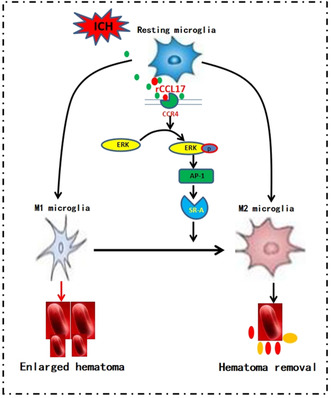

## 1 Introduction

Intracerebral hemorrhage (ICH) is a severe stroke with high morbidity and mortality rates, and persistent hematoma worsens outcomes, emphasizing the need for more effective treatments [1, 2, 3]. Current interventions mainly address symptoms rather than the underlying pathophysiological processes [4, 5, 6]. While surgical techniques like early hematoma removal have shown promise, reducing hematoma expansion remains a challenge [7, 8]. Hematoma clearance is crucial for mitigating secondary brain injury [9, 10, 11].

Our previous work has demonstrated that microglia, the brain's resident immune cells, are crucial mediators of neuroinflammation following ICH [12, 13]. These cells exhibit remarkable plasticity, transitioning between pro‐inflammatory (M1) and anti‐inflammatory (M2) phenotypes [14, 15]. We and others have shown that the M2 phenotype is particularly important for hematoma clearance, making microglial polarization an attractive therapeutic target [16, 17]. Our recent studies [12] identified key mechanisms regulating microglial phenotype transitions, which are crucial for developing targeted therapies [18, 19].

Building on our previous findings [12, 13], we identified the CCL17/CCR4 signaling axis as a critical regulator of immune cell function and polarization. While our recent work [20] demonstrated this axis's role in driving macrophage polarization toward a reparative phenotype [21, 22, 23, 24], the specific mechanisms by which CCL17/CCR4 signaling influences microglial polarization during ICH require further investigation.

The ERK/AP1/SRA signaling pathway emerges as a potential mechanistic link in this process. ERK, a downstream effector of CCR4 [25], orchestrates cellular responses to external stimuli. AP1, activated by ERK, functions as a crucial transcription factor regulating immune responses and inflammatory gene expression [26]. Scavenger receptor A (SRA), whose expression is controlled by AP1, plays a vital role in phagocytosis and debris clearance [27, 28, 29]. Our previous studies suggest that understanding how CCR4 activation impacts this pathway could reveal new therapeutic strategies for ICH.

Based on our preliminary data and published findings, we hypothesize that CCL17/CCR4 signaling modulates the ERK/AP1/SRA pathway to promote M2 polarization, thereby facilitating hematoma clearance and reducing secondary brain injury. This study aims to: (1) Further characterize CCR4 expression patterns in microglia post‐ICH; (2) Delineate the downstream ERK/AP1/SRA signaling cascade; (3) Evaluate CCL17/CCR4 signaling effects on microglial phenotype and hematoma resolution; (4) Assess the therapeutic potential of targeting this axis in experimental ICH. This investigation extends our previous work on CCL17/CCR4‐mediated hematoma resolution and provides new insights into potential therapeutic strategies for ICH treatment (Figure S2).

## 2 Materials and Methods

### 2.1 Animals and ICH Model

All animal procedures were approved by the Ethics Committee for Laboratory Animal Experiments of Fudan University (Approval Number: 2021JS Huashan Hospital‐510) and conducted in accordance with the ARRIVE 2.0 guidelines. The study used adult male C57BL/6 mice (8–10 weeks old, weighing 22–28 g) obtained from Zhejiang Vital River Laboratory Animal Technology Co. Ltd. Animals were housed in a specific‐pathogen‐free facility under controlled conditions (temperature: 22°C ± 2°C; humidity: 55% ± 5%) with a 12‐h light/dark cycle (lights on 7:00–19:00). Standard laboratory chow and water were available ad libitum, with 4–5 mice housed per ventilated cage containing corn cob bedding. Regular health monitoring was performed by veterinary staff.

Sample size was based on our previous studies [12]. An additional 20% was included to account for potential mortality. Animals meeting the inclusion criteria (male C57BL/6 mice, 8–10 weeks old, 22–28 g weight, normal baseline behavioral scores) were randomly assigned to experimental groups using SPSS (version 18.0), with randomization stratified by body weight. Each animal received a unique identification code.

To ensure experimental rigor, three independent investigators participated in the study. Investigator A performed randomization and treatment administration, Investigator B conducted surgical procedures and behavioral testing, and Investigator C analyzed the data. Treatment codes were revealed only after all data collection was completed. Exclusion criteria included surgical complications, death within 24 h post‐surgery, infection or health complications, and extreme outliers in behavioral tests. Predefined endpoint criteria were established, including severe neurological deficits, weight loss exceeding 20%, signs of severe distress, or complications. Animals were monitored daily for these endpoints to ensure humane experimental conditions.

The ICH model was induced by intrastriatal autologous blood injection, as previously described [12]. Briefly, mice were anesthetized with an intraperitoneal injection of ketamine (100 mg/kg) and xylazine (10 mg/kg). Following positioning in a stereotaxic frame, a small burr hole was drilled in the skull, and a 26‐gauge Hamilton syringe needle was inserted into the right striatum (coordinates: 0.2 mm anterior, 2.3 mm lateral to the bregma, and 3.5 mm ventral to the skull). Autologous blood (30 μL) was infused at a rate of 2 μL/min using an infusion pump. To prevent reflux, the needle was left in place for an additional 10 min post‐injection. Sham‐operated mice underwent the same procedure without blood infusion.

### 2.2 Experimental Design

This study was divided into five distinct experiments to investigate the role of CCL17 and its receptor CCR4 in the pathophysiology of intracerebral hemorrhage (ICH) and to explore the potential therapeutic effects of recombinant CCL17 (rCCL17), along with the underlying mechanisms. The experimental groups and interventions are outlined below.

#### 2.2.1 Experiment 1: Temporal Expression of Endogenous CCR4 and Downstream Signaling Molecules After ICH

Mice were randomly assigned to seven groups (*n* = 6/group): sham, ICH‐6 h, ICH‐12 h, ICH‐24 h, ICH‐72 h, ICH‐5 day, and ICH‐7 day. Brain samples were collected at the indicated time points for western blot analysis of CCR4, ERK1/2, p‐ERK1/2, AP1, and SRA expression. Additionally, four mice per group (sham and ICH‐72 h) were used for immunofluorescence staining to examine the cellular localization of SRA and CCR4 in microglia, astrocytes, and neurons.

#### 2.2.2 Experiment 2: Impact of CCR4 CRISPR Knockdown or Overexpression on ICH Outcomes

Mice were randomly assigned to six groups (*n* = 6/group): wildtype + Sham, CCR4^−/−^ + Sham, CCR4^overexpress^ + Sham, wildtype + ICH, CCR4^−/−^ + ICH, and CCR4^overexpress^  + ICH. Neurobehavioral tests and hematoma volume assessments were conducted on Day 3 post‐ICH.

#### 2.2.3 Experiment 3: Effects of rCCL17 Treatment on Neurological Deficits, Brain Edema, Hematoma Volume, and Microglial/Macrophage Polarization Post‐ICH

Mice were divided into three groups (*n* = 6/group): sham, ICH + vehicle, and ICH  + rCCL17. Neurobehavioral tests, brain water content measurements, and hematoma volume assessments were performed on Days 1, 3, and 7 post‐ICH. Additionally, four mice per group at 72 h post‐ICH were used for immunofluorescence staining to assess the cellular localization of SRA and microglial/macrophage polarization. Western blot analysis was also conducted to assess microglial/macrophage polarization and SRA expression.

#### 2.2.4 Experiment 4: Evaluating CCL17's Protective Effects Through Its Receptor CCR4 Post‐ICH

Mice were randomly assigned to five groups (*n* = 6/group): ICH + CCR4^−/−^ + Vehicle, ICH + CCR4^overexpress^  + Vehicle, WT  + ICH + rCCL17, ICH + CCR4^−/−^  + rCCL17, and ICH + CCR4^overexpress^ + rCCL17. Neurobehavioral tests and hematoma volume assessments were performed on day 3 post‐ICH.

#### 2.2.5 Experiment 5: Examining the Activation of the ERK/AP‐1/SRA Pathway by CCL17/CCR4 Signaling Post‐ICH

Mice were divided into five groups (*n* = 6/group): sham, ICH + vehicle, ICH + rCCL17, ICH  + rCCL17 + DMSO, and ICH + rCCL17 + U0126. Western blot analysis was conducted on day 3 post‐ICH to assess CCR4 expression, the ERK/AP‐1/SRA pathway, and microglial/macrophage polarization.

### 2.3 Drug Administration and CRISPR‐Mediated CCR4 Knockdown and Overexpression

Recombinant mouse CCL17 (14,013, LSBio, WA) was dissolved in 5% dimethyl sulfoxide (DMSO) and administered intranasally at a concentration of 30 μg/kg. The intranasal administration protocol involved delivering a total volume of 20 μL of rCCL17, with one drop (5 μL/drop) applied every 5 min, alternating between the two nares over a 20‐min period. This daily intranasal delivery was performed for 1 day. For ERK pathway inhibition, U0126 (sc‐222,395, Santa Cruz Biotechnology, USA), an ERK inhibitor, was dissolved in 5% DMSO and administered intraperitoneally at a dose of 30 mg/kg, 1 h post‐ICH.

For genetic manipulation studies, CCR4 CRISPR knockout plasmids (r) (GemPharmatech, China) and CCR4 lentiviral overexpression virus (KeyGEN BioTECH, China) were administered via intracerebroventricular injection (i.c.v.) 48 h before ICH induction, following the manufacturer's instructions. The CCR4 CRISPR knockout plasmids were administered at a concentration of 1 μg/μL with a total volume of 2 μL, while the CCR4 lentiviral overexpression virus was delivered at 1 × 10^6^9^ TU/mL with a total volume of 2 μL. The stereotaxic coordinates for the lateral ventricle were precisely defined as 0.3 mm posterior, 1.0 mm lateral to the bregma, and 2.3 mm ventral to the skull surface. Both the CRISPR knockout plasmids (r) and viral suspension were administered at a controlled rate of 0.1 μL/min [12]. Following genetic manipulation, all subsequent procedures adhered to the standardized ICH model protocol. A detailed flow diagram illustrating the experimental timeline and procedures has been included to enhance clarity (Figure S1).

### 2.4 Neurobehavioral Assessment

Neurobehavioral tests, including the modified Garcia test, corner turn test, and forelimb placement test, were conducted by an investigator blinded to the experimental groups on Days 1 and 3 post‐ICH, as previously described [12]. The modified Garcia test included seven subtests evaluating spontaneous activity, limb movement symmetry, forepaw outstretching, climbing, body proprioception, response to vibrissae touch, and lateral turning. Each subtest was scored from 0 to 3, with a maximum total score of 21. For the corner turn test, mice were placed facing a 30° corner, and the number of right turns out of 10 trials was recorded. For the forelimb placement test, the left forelimb placement was calculated as left forelimb placement/(left forelimb placement + right forelimb placement) × 100%.

### 2.5 Brain Water Content Measurement

Brain water content was measured on Days 1 and 3 post‐ICH using the wet‐dry method [13]. Mice were deeply anesthetized, decapitated, and their brains quickly removed and divided into five parts: ipsilateral and contralateral cortex, ipsilateral and contralateral basal ganglia, and cerebellum. Each brain sample was immediately weighed on an electronic analytical balance (Mettler Toledo, Columbus, OH, USA) to obtain the wet weight. The samples were then dried in an oven at 100°C for 24 h and reweighed to obtain the dry weight. The percentage of brain water content was calculated as [(wet weight‐dry weight)/wet weight] × 100%.

### 2.6 Immunofluorescence Staining

Mice were deeply anesthetized and transcardially perfused with ice‐cold PBS followed by 4% paraformaldehyde on Day 3 post‐ICH [13]. Brains were removed, post‐fixed overnight in 4% paraformaldehyde, and cryoprotected in 30% sucrose for 48 h. Coronal brain sections (10 μm) were cut using a cryostat (Leica Microsystems, Wetzlar, Germany) and stored at −80°C until further use. For immunofluorescence staining, sections were washed with PBS, blocked with 5% normal donkey serum in PBS containing 0.3% Triton X‐100 for 1 h at room temperature, and incubated overnight at 4°C with the following primary antibodies: goat anti‐Iba1 (1:500, Abcam), rabbit anti‐CD16/32 (1:200, BD Biosciences), mouse anti‐CD206 (1:200, Bio‐Rad), and mouse anti‐SRA (1:200, XY24655‐1, XYbscience). After washing with PBS, sections were incubated with the corresponding Alexa Fluor 488‐, 594‐, or 647‐conjugated secondary antibodies (1:1000, Invitrogen) for 1 h at room temperature. Nuclei were counterstained with DAPI (1:10,000, Invitrogen). Sections were mounted on slides and cover slipped with Fluoromount‐G (SouthernBiotech). Protein bands were visualized using enhanced chemiluminescence reagents and quantified with ImageJ software.

### 2.7 Western Blot Analysis

Mice were deeply anesthetized and transcardially perfused with ice‐cold PBS on Day 3 post‐ICH [13]. The ipsilateral striatum was dissected and homogenized in RIPA lysis buffer containing protease and phosphatase inhibitors. Equal amounts of protein (40 μg) were separated by SDS‐PAGE and transferred onto PVDF membranes. The membranes were blocked with 5% nonfat milk for 1 h at room temperature and incubated overnight at 4°C with the following primary antibodies: rabbit anti‐CCR4 (1:1000, GTX53474, GeneTex), rabbit anti‐ERK1/2 (1:1000, Cell Signaling Technology), rabbit anti‐phospho‐ERK1/2 (1:1000, Cell Signaling Technology), CD16 (1:100, Santa Cruz Biotechnology), CD206 (1:100, Santa Cruz Biotechnology), AP1 (1:100, Novus Biologicals), rabbit anti‐SRA (1:1000, Abcam), and mouse anti‐β‐actin (1:5000, Sigma‐Aldrich). After washing with TBST, the membranes were incubated with horseradish peroxidase‐conjugated secondary antibodies (1:5000, Jackson ImmunoResearch) for 1 h at room temperature. Immunoreactive bands were visualized using an enhanced chemiluminescence substrate (Thermo Fisher Scientific) and quantified using ImageJ software.

### 2.8 Hematoma Volume Measurement and Hemoglobin Assay

Hematoma volume was assessed on Days 1, 3 and 7 post‐ICH using spectrophotometric quantification, as previously described [12]. Hemoglobin content in brain tissue was measured using Drabkin's reagent, following the manufacturer's instructions (Sigma‐Aldrich). Six mice per group were used for hematoma volume measurement and hemoglobin assay.

### 2.9 Statistical Analysis

Data are presented as the mean ± standard deviation (SD). Statistical analyses were conducted using GraphPad Prism 8 software (GraphPad Software Inc., San Diego, CA, USA). Because the sample size of each group is small, the Shapiro–Wilk test is first used to determine whether the data follow a normal distribution. If they do follow a normal distribution, one‐way ANOVA is performed, and pairwise comparisons among multiple groups are carried out using the Tukey HSD (Honestly Significant Difference) method. A two independent samples *t*‐test was used for comparisons between two groups. A *p* value of < 0.05 was considered statistically significant.

## 3 Results

### 3.1 CCR4 And Its Downstream Signaling Molecules Exhibit Dynamic Regulation Following ICH

**FIGURE 1:**
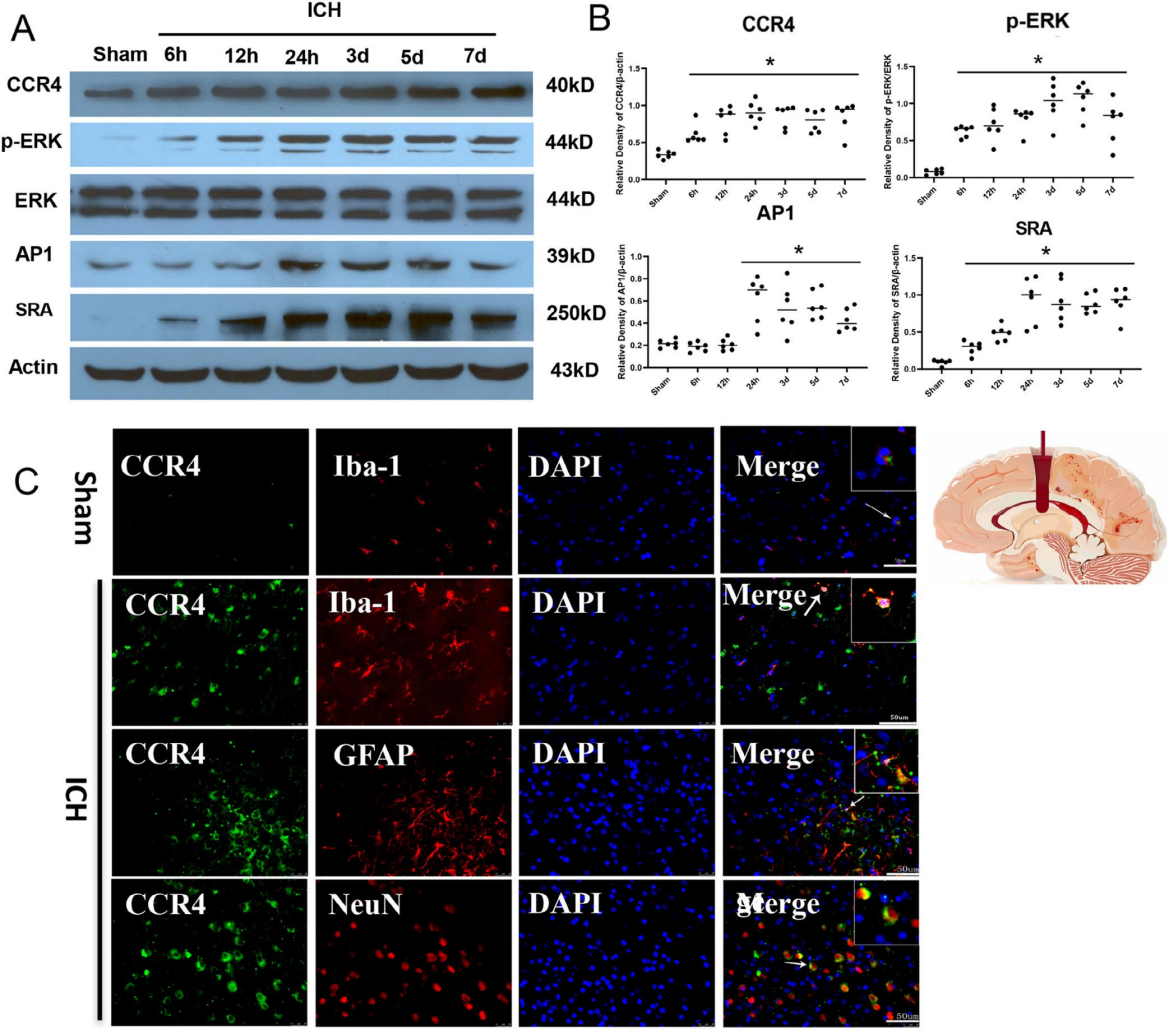
The time course and activation of the CCL17/CCR4 signaling pathway following intracerebral hemorrhage (ICH) in mice. (A) Representative Western blot images demonstrate the protein expression levels of CCR4, p‐ERK, ERK, AP‐1, and SRA in the ipsilateral hemisphere at different time points (sham; 6h, 12h, 24 h; 3d, 5d, and 7 day) after ICH. (B) Quantitative analysis of the relative density of CCR4, p‐ERK, AP‐1, and SRA protein expression (*n* = 6/group). (C) Immunofluorescence shows the cellular localization of CCR4 following ICH (*n* = 4/group). *p < 0.05 versus sham, Mean ± SD, One‐way ANOVA, Tukey test.

We first analyzed the temporal expression patterns of CCR4 and its associated signaling proteins in the ipsilateral hemisphere of ICH mice using western blotting. CCR4 levels began to rise at 6 h post‐ICH and remained elevated for up to 7 days, compared to the sham group (Figure 1A,B). Phosphorylated ERK (p‐ERK) followed a similar trajectory, with significantly higher levels observed from 6 h to 7 days after ICH (Figure 1B). The expression of AP‐1, a transcription factor downstream of ERK, increased starting at 12 h and persisted until 7 days post‐ICH (Figure 1B). Additionally, SR‐A, a scavenger receptor involved in microglial/macrophage functions, showed elevated levels from 6 h to 7 days post‐ICH (Figure 1B). These findings suggest that the CCR4 signaling cascade is activated in a time‐dependent manner following experimental ICH. Immunofluorescence staining revealed that CCR4 is expressed in microglia, astrocytes, and neurons, with a more prominent expression observed in microglia (Figure 1C).

### 3.2 CCR4 Deficiency and Overexpression Impact Hematoma Volume and Neurological Outcomes After ICH

**FIGURE 2:**
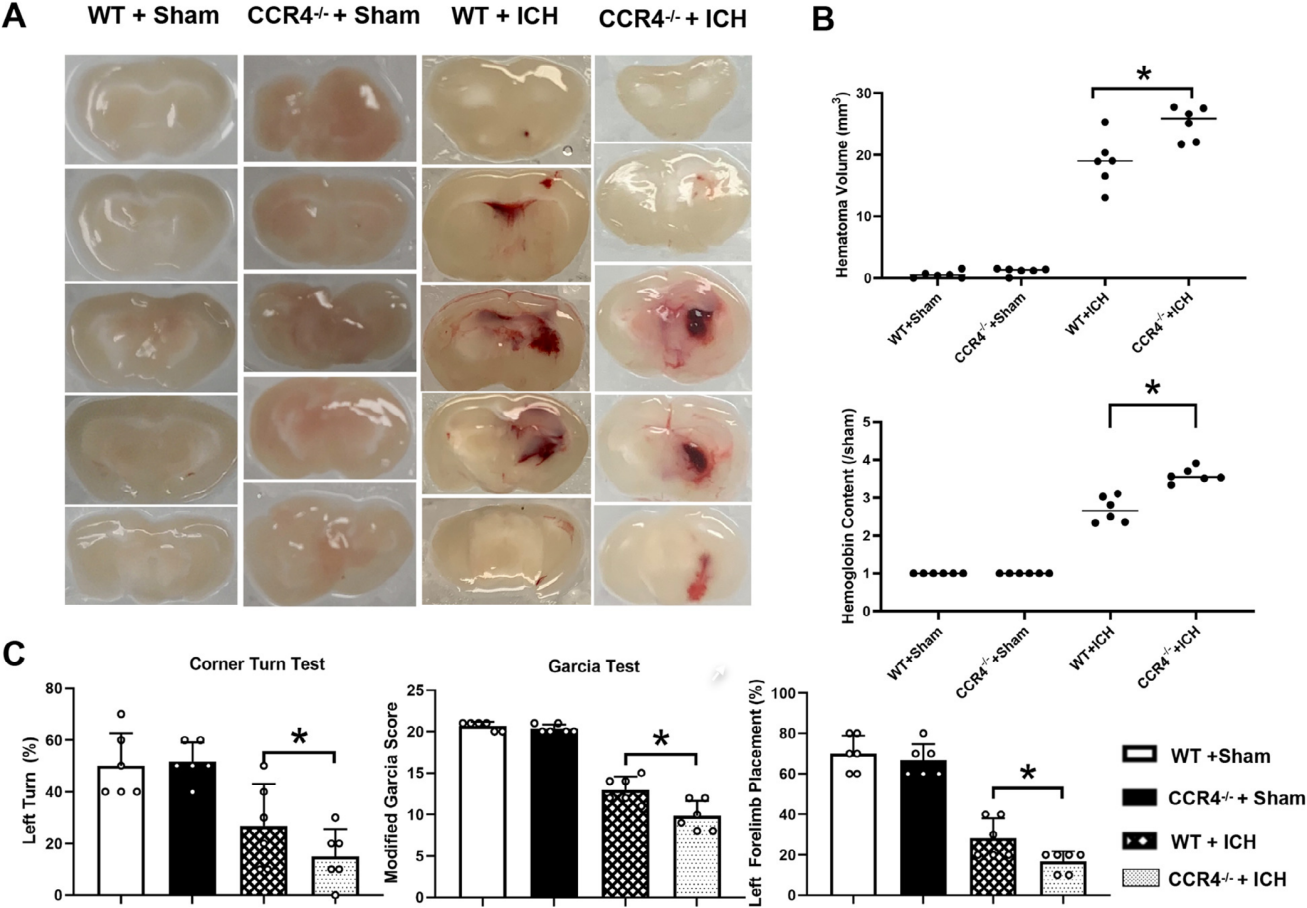
CCR4 deficiency increases hematoma volume and exacerbates neurological deficits after ICH. (A) brain sections from CCR4^−/−^ ICH mice exhibited larger hematoma volumes compared to WT + ICH mice. (B) Quantitative analysis confirmed that CCR4^−/−^ ICH mice had significantly larger hematoma volumes compared to WT + ICH mice. Furthermore, the hemoglobin content in the brain tissue was also higher in CCR4^−/−^ ICH mice. (C) CCR4^−/−^ ICH mice performed worse in the corner turn test, Garcia test, and limb placement test. CCR4^−/−^: CCR4 CRISPR knockout, WT: Wildtype, *n* = 6/group, *p < 0.05 versus WT + ICH, Mean ± SD, two independent samples *t*‐test.

To investigate the role of CCR4 in ICH pathology, we utilized both CCR4 CRISPR knockout mice (CCR4^−/−^) and CCR4 lentiviral overexpression mice (CCR4^overexpress^), comparing them to wild‐type (WT) controls. Hematoma volume at 72 h post‐ICH was significantly larger in CCR4^−/−^ mice compared to WT controls (Figure 2A,B). Similarly, hemoglobin content, an indicator of hematoma size, was higher in the ipsilateral hemisphere of CCR4^−/−^ mice, further suggesting that CCR4 deficiency exacerbates hematoma expansion following ICH.

Functionally, CCR4^−/−^ mice exhibited more severe neurological impairments than WT mice, as assessed by the Corner Turn test, Garcia test, and Limb Placement test (Figure 2C). CCR4^−/−^ mice showed a significantly higher percentage of left turns in the Corner Turn test, a lower Garcia test score, and reduced Limb Placement scores, indicating worse overall neurological function and more severe sensorimotor deficits. In contrast, sham‐operated animals showed no significant differences between genotypes in these behavioral tests. These findings indicate that CCR4 deficiency worsens ICH‐induced brain injury and neurological dysfunction, suggesting a protective role for endogenous CCR4 signaling in ICH.

**FIGURE 3:**
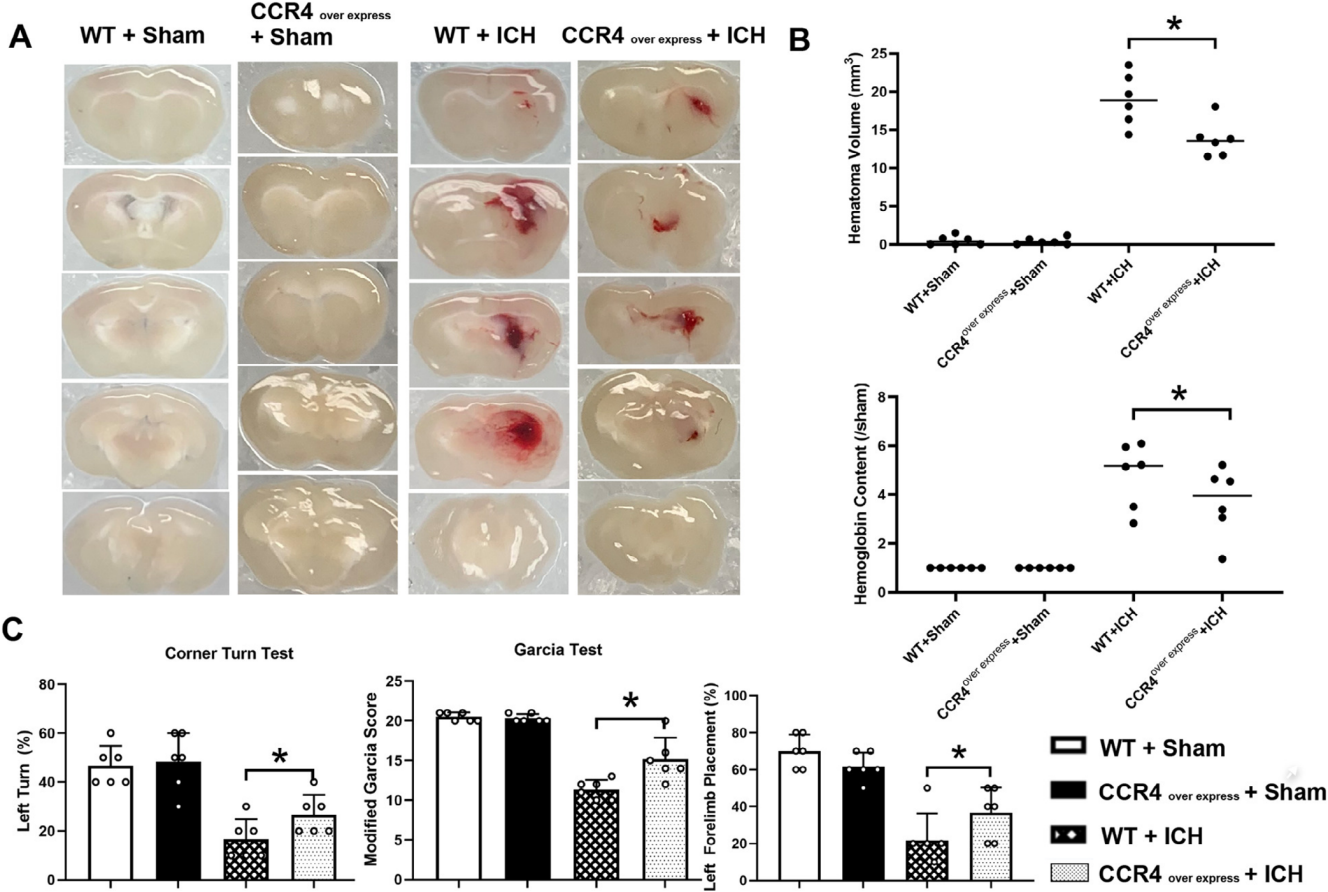
CCR4 overexpression reduces hematoma volume and mitigates neurological deficits after ICH. (A) brain sections from CCR4^overexpress^ ICH mice exhibited smaller hematoma volumes compared to WT + ICH mice. (B) Quantitative analysis confirmed that CCR4^overexpress^ ICH mice had significantly smaller hematoma volumes compared to WT + ICH mice. Furthermore, the hemoglobin content in the brain tissue was also lower in CCR4^overexpress^ ICH mice. (C) CCR4^overexpress^ ICH mice performed better in the corner turn test, Garcia test, and limb placement test. CCR4^overexpress^: CCR4 CRISPR overexpression, WT: Wildtype, *n* = 6/group, *p < 0.05 versus WT + ICH, Mean ± SD, two independent samples *t*‐test.

Conversely, CCR4 overexpression in CCR4^overexpress^ mice at 72 h post‐ICH resulted in significantly reduced hematoma volume and improved neurological outcomes compared to sham controls (Figure 3A–C). These results further support the protective role of CCR4 signaling in mitigating brain injury and neurological deficits after ICH.

### 3.3 rCCL17 Treatment Enhances Neurological Function, Reduces Hematoma Volume, Decreases Brain Edema, and Modulates Microglial Polarization After ICH

To explore the therapeutic potential of rCCL17 in ICH, we administered recombinant CCL17 (rCCL17), a CCR4 ligand, intranasally to mice 1 h post‐ICH and evaluated its effects on neurological function, hematoma progression, brain edema, and microglial polarization.

**FIGURE 4:**
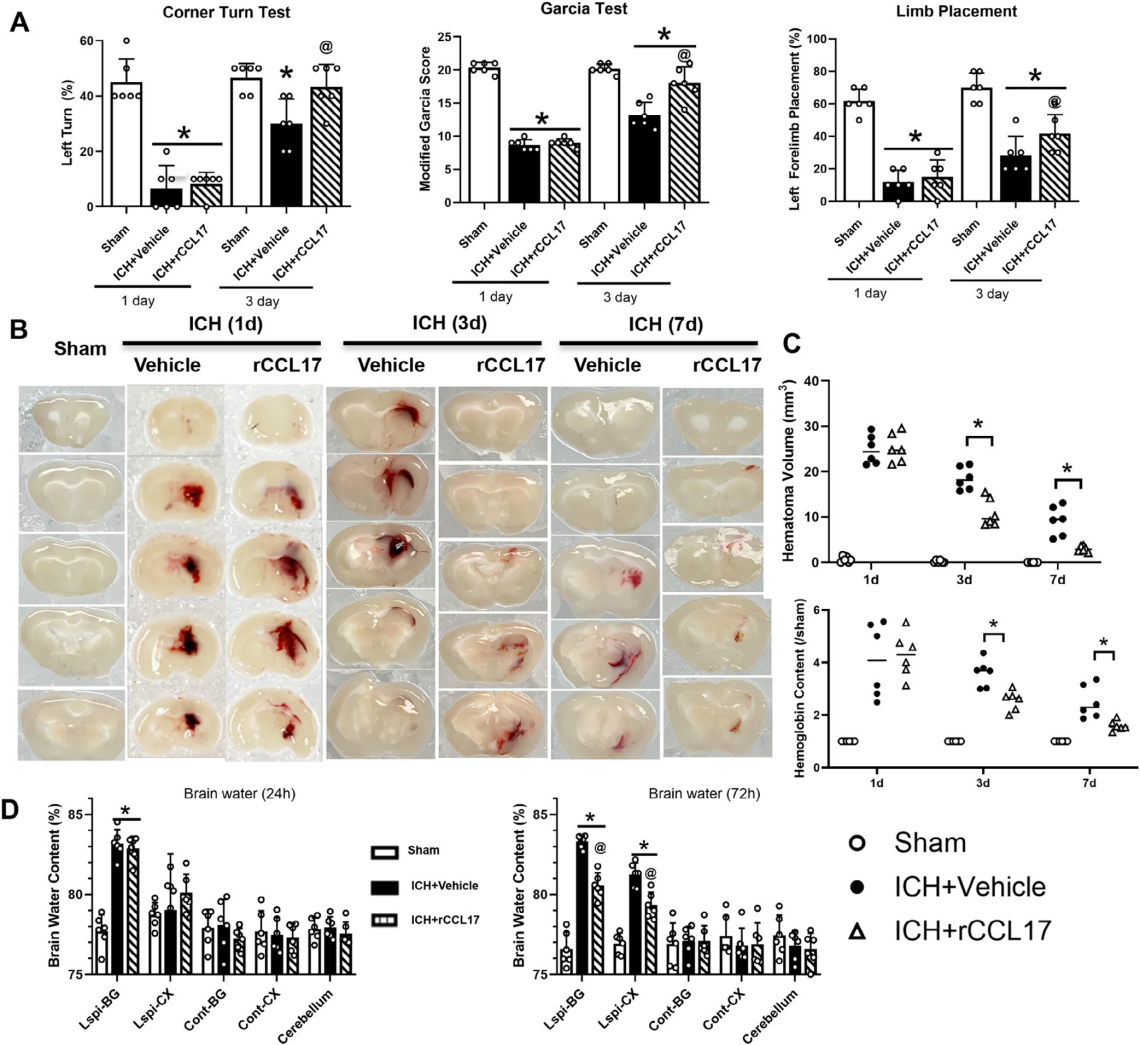
rCCL17 treatment enhances neurological function, reduces hematoma volume, and decreases brain edema post‐ICH. (A) Behavioral tests were conducted at 1 and 3 days post‐ICH. (B) Representative brain sections showing hematoma volumes at 1 day (1d), 3 days (3d), and 7 days (7d) post‐ICH. rCCL17 treatment visibly reduced hematoma size compared to vehicle‐treated ICH mice at 3d and 7d. (C) Quantitative analysis of hematoma volumes Hemoglobin content confirming that rCCL17 treatment significantly decreased hematoma volumes at 3d, and 7d post‐ICH compared to vehicle‐treated controls. (D) Brain water content was measured at 24 and 72 h after ICH. *n* = 6/group, *p < 0.05 versus sham, @ *p* < 0.05 versus ICH + Vehicle, Mean ± SD, One‐way ANOVA, Tukey test.

Neurological assessment at 3 days post‐ICH revealed significant improvements in rCCL17‐treated mice. The Corner Turn test showed a marked reduction in left turns compared to vehicle‐treated ICH mice (Figure 4A), indicating better sensorimotor function. Similarly, higher Garcia test and Limb Placement scores in the rCCL17‐treated groups suggested improved overall neurological performance and sensorimotor integration (Figure 4A).

Histological analysis showed that rCCL17 treatment significantly reduced hematoma volume at both 3 and 7 days post‐ICH, as visualized by brain sections and confirmed by quantitative analysis (Figure 4B,C). Additionally, rCCL17 treatment significantly lowered brain water content, indicating reduced brain edema, at 3 days post‐ICH (Figure 4D).

**FIGURE 5:**
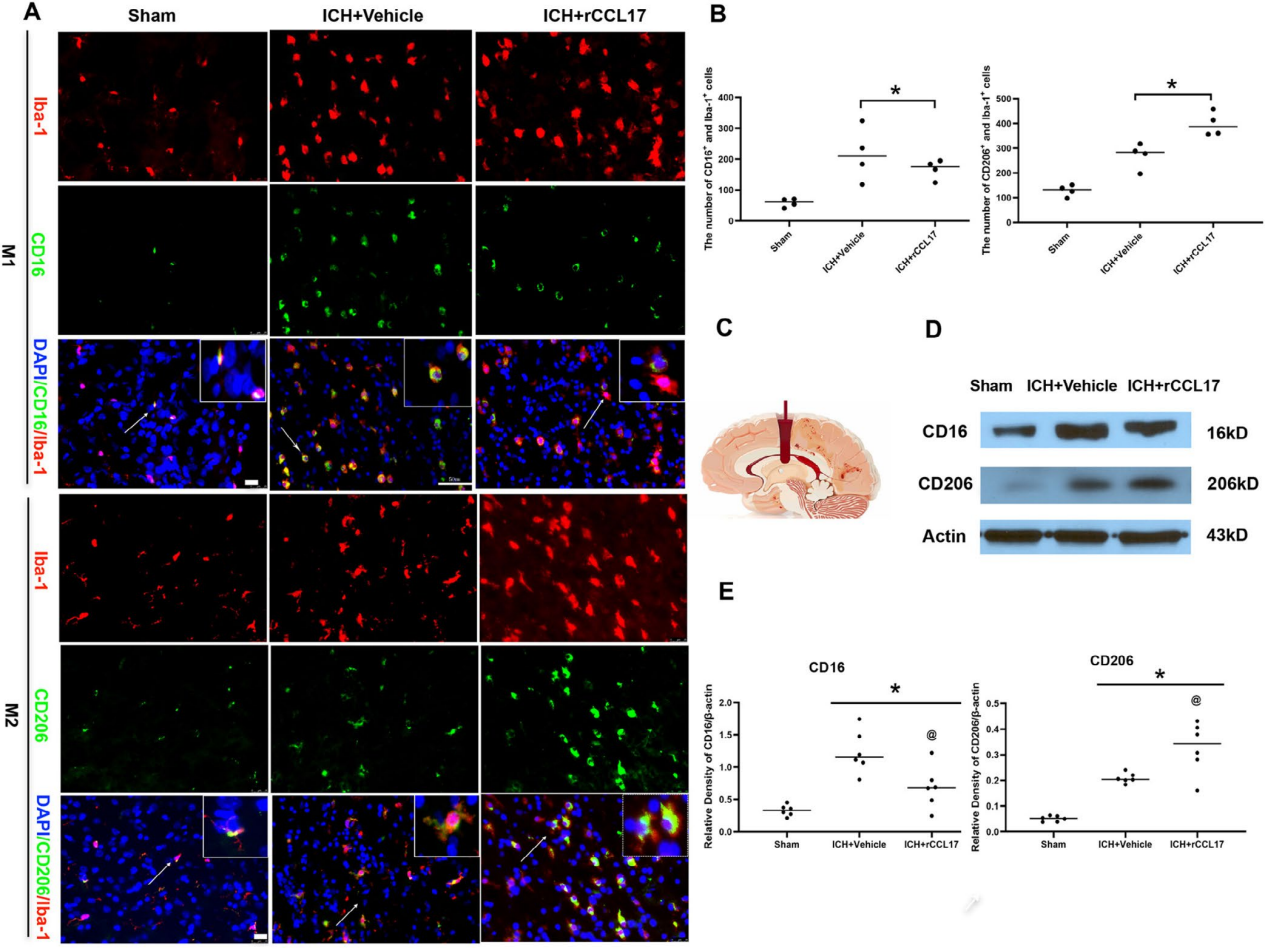
rCCL17 promotes M2 microglial polarization and suppresses M1 activation in the perihematomal region at 3 days post‐ICH. (A) Immunofluorescence staining of the perihematomal region in sham, ICH + Vehicle, and ICH + rCCL17 mice. The images show the expression of the M1 microglia marker CD16 (green) and the M2 microglia marker CD206 (green), DAPI (blue), Iba‐1 (red), and CD16/CD206 (green). (B) Quantitative analysis of the number of CD206^+^ and CD16 positive microglia cells in the perihematomal region. rCCL17 treatment significantly increased the number of CD206^+^ cells (M2 microglia) and decreased the number of CD16^+^ cells (M1 microglia) compared to vehicle‐treated ICH mice. (C) Schematic diagram of the intracerebral hemorrhage model. (D) Western blot analysis of CD16 and CD206 expression in the ipsilateral hemisphere of sham, ICH + Vehicle, and ICH + rCCL17 mice. (E) Quantitative analysis of CD16 and CD206 protein expression based on the Western blot results. *n* = 4/group for immunofluorescence staining, *n* = 6/group for western blot. *p < 0.05 versus sham, @ *p* < 0.05 versus ICH + Vehicle, Mean ± SD, One‐way ANOVA, Tukey test.

At the cellular level, rCCL17 treatment promoted a shift in microglial polarization toward an anti‐inflammatory M2 phenotype. Immunofluorescence staining revealed a significant increase in IBA1^+^CD206^+^ (M2) cells and a decrease in IBA1^+^CD16^+^ (M1) cells in the perihematomal region of rCCL17‐treated mice compared to vehicle‐treated controls (Figure 5A–C). Western blot analysis further confirmed these findings, showing increased CD206 and decreased CD16 expression in rCCL17‐treated mice (Figure 5D,E).

**FIGURE 6:**
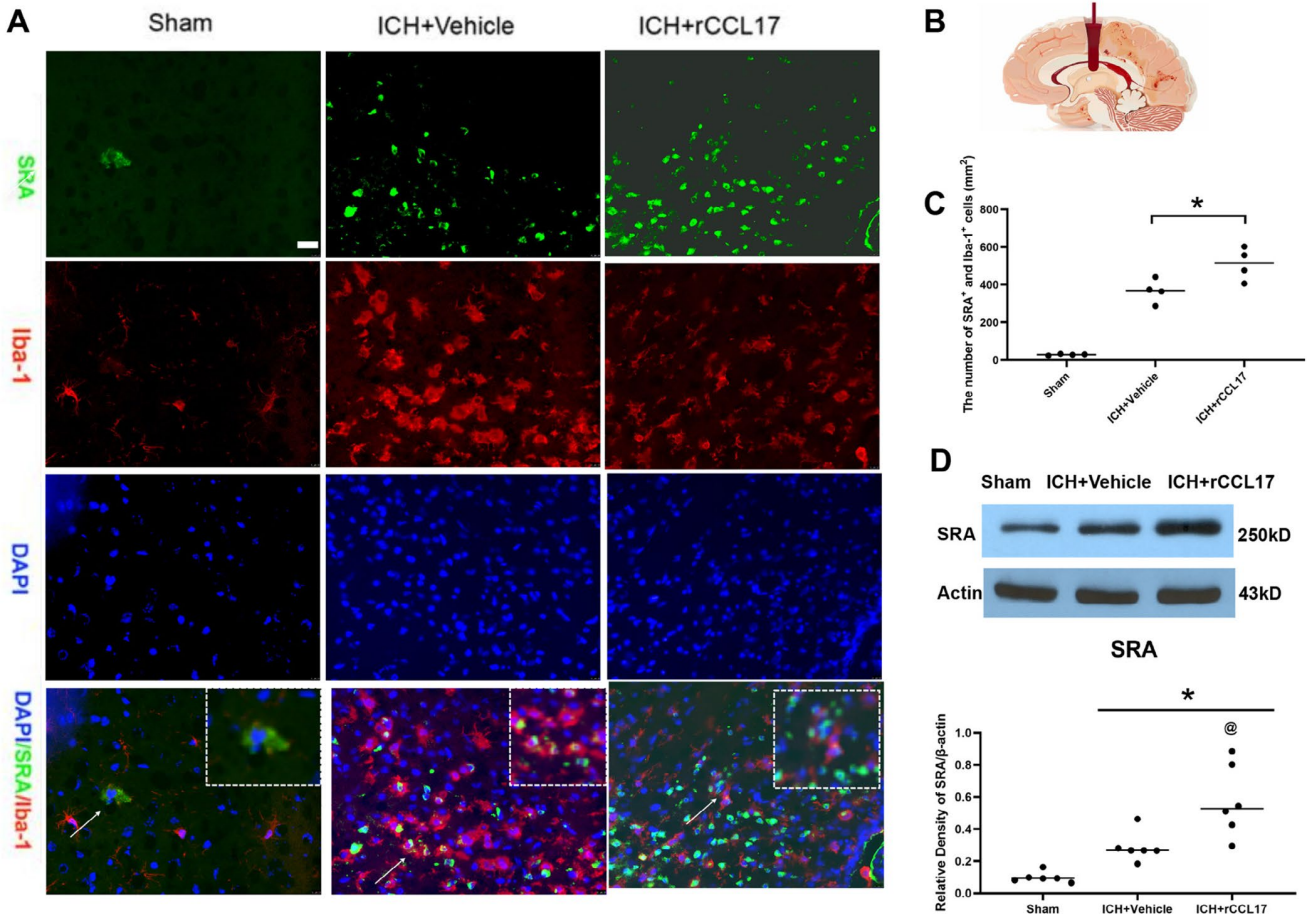
rCCL17 enhances SRA expression on microglia after ICH. (A) Immunofluorescence staining of the perihematomal region in sham, ICH + Vehicle, and ICH + rCCL17 mice. The images show the expression of the SRA and microglia, DAPI (blue), Iba‐1 (red), and SRA (green). (B) schematic diagram of the intracerebral hemorrhage model. (C) Quantitative analysis of the number of SRA positive microglia cells in the perihematomal region. rCCL17 treatment significantly increased the number of SRA^+^ cells compared to vehicle‐treated ICH mice. (D) Western blot and Quantitative analysis of SRA expression in the ipsilateral hemisphere of sham, ICH + Vehicle, and ICH + rCCL17 mice. *n* = 4/group for immunofluorescence staining, *n* = 6/group for western blot. *p < 0.05 versus sham, @ *p* < 0.05 versus ICH + Vehicle, Mean ± SD, One‐way ANOVA, Tukey test.

Furthermore, rCCL17 treatment enhanced SRA expression on microglia, a key receptor for phagocytosis. Immunofluorescence staining showed a significant increase in IBA1^+^SRA^+^ cells in the perihematomal region of rCCL17‐treated mice compared to controls (Figure 6A–C). This was further supported by western blot analysis, which confirmed upregulated SRA expression in rCCL17‐treated mice (Figure 6D). These findings suggest that rCCL17 enhances microglial phagocytosis and supports hematoma clearance by promoting both M2 polarization and increased SRA expression.

### 3.4 CCL17 Mediates Protective Effects via the CCR4 Receptor After ICH

**FIGURE 7:**
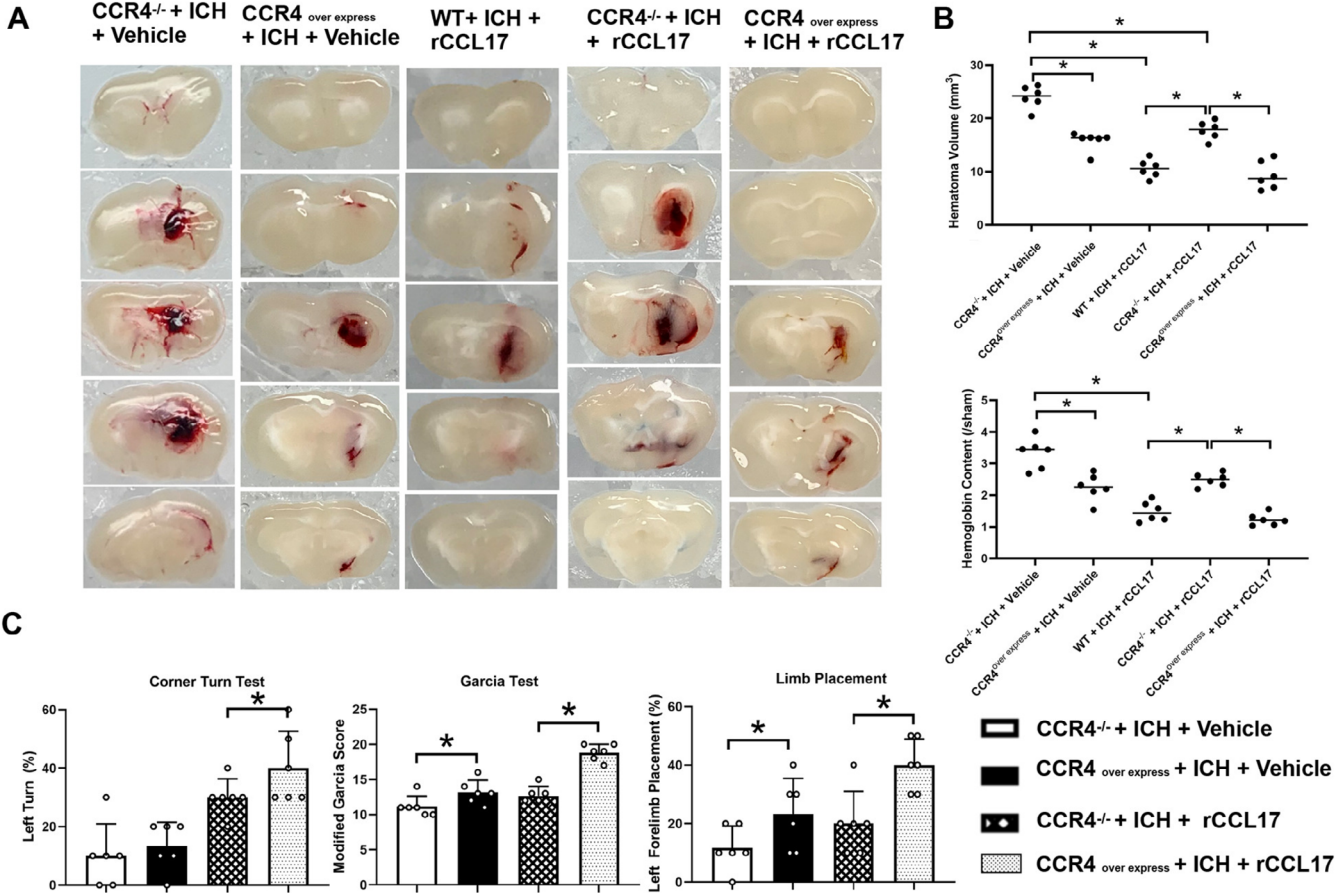
CCL17 Exerts Protective Effects via the CCR4 Receptor After ICH. (A) Representative brain sections showing hematoma volumes in CCR4^−/−^ and CCR4^overexpress^ ICH mice treated with vehicle or rCCL17. (B) Quantitative analysis confirmed that rCCL17 treatment significantly reduced both hematoma volumes and hemoglobin content in CCR4^−/−^ and CCR4‐overexpressing ICH mice compared with vehicle‐treated controls. Furthermore, the CCR4^−/−^ + ICH + rCCL17 group demonstrated impaired hematoma resolution compared with the WT + ICH + rCCL17 group. (C) Behavioral tests were conducted to assess neurological function in CCR4^−/−^ and CCR4^overexpress^ ICH mice treated with vehicle or rCCL17. CCR4^−/−^: CCR4 CRISPR knockout, CCR4^overexpress^: CCR4 CRISPR overexpression, *n* = 6/group, *p < 0.05 versus CCR4^−/−^ + ICH + Vehicle and versus CCR4^overexpress^ + ICH + Vehicle versus WT + ICH + rCCL17 group, Mean ± SD, two independent samples *t*‐test.

To investigate whether rCCL17's therapeutic effects are mediated through CCR4, we administered rCCL17 to CCR4‐overexpressing (CCR4^overexpress^) mice and compared outcomes with CCR4 CRISPR knockout (CCR4^−/−^) and vehicle‐treated mice after ICH. Brain sections showed significantly smaller hematoma volumes in rCCL17‐treated CCR4^overexpress^ mice compared to CCR4^−/−^ and vehicle‐treated groups at 72 h post‐ICH (Figure 7A,B). Quantification confirmed a greater reduction in hematoma volume in the rCCL17‐treated CCR4^overexpress^ group (Figure 7B), with lower hemoglobin content in the ipsilateral hemisphere compared to the other groups (Figure 7B). These results suggest that CCR4 overexpression enhances the hematoma‐resolving effects of rCCL17. We have conducted additional experiments specifically comparing the hematoma sizes between rCCL17 + CCR4^−/−^ + ICH group and WT + ICH + rCCL17 group. Our analysis reveals significant differences in hematoma volume between these groups. Specifically, we found that the CCR4^−/−^ + ICH + rCCL17 group exhibited impaired hematoma resolution compared to the WT + ICH + rCCL17 group. These differences were statistically significant (*p* ≤ 0.05) (Figure 7A,B).

Functionally, rCCL17 treatment significantly improved sensorimotor function in CCR4^overexpress^ mice, as shown by a greater decrease in the percentage of left turns in the Corner Turn test and higher scores in the Garcia and Limb Placement tests compared to CCR4^−/−^ and vehicle‐treated mice (Figure 7C). These findings further support the role of CCR4 signaling in mediating the therapeutic effects of rCCL17 on functional recovery.

Interestingly, vehicle‐treated CCR4^overexpress^ mice also showed some improvement in hematoma volume, hemoglobin content, and neurological function compared to vehicle‐treated CCR4^−/−^ mice (Figure 7A‐C), suggesting an inherent protective effect of CCR4 overexpression in ICH.

### 3.5 rCCL17 Promotes Microglial Polarization via ERK/AP‐1 Signaling and SRA Upregulation in the Perihematomal Region After ICH

**FIGURE 8:**
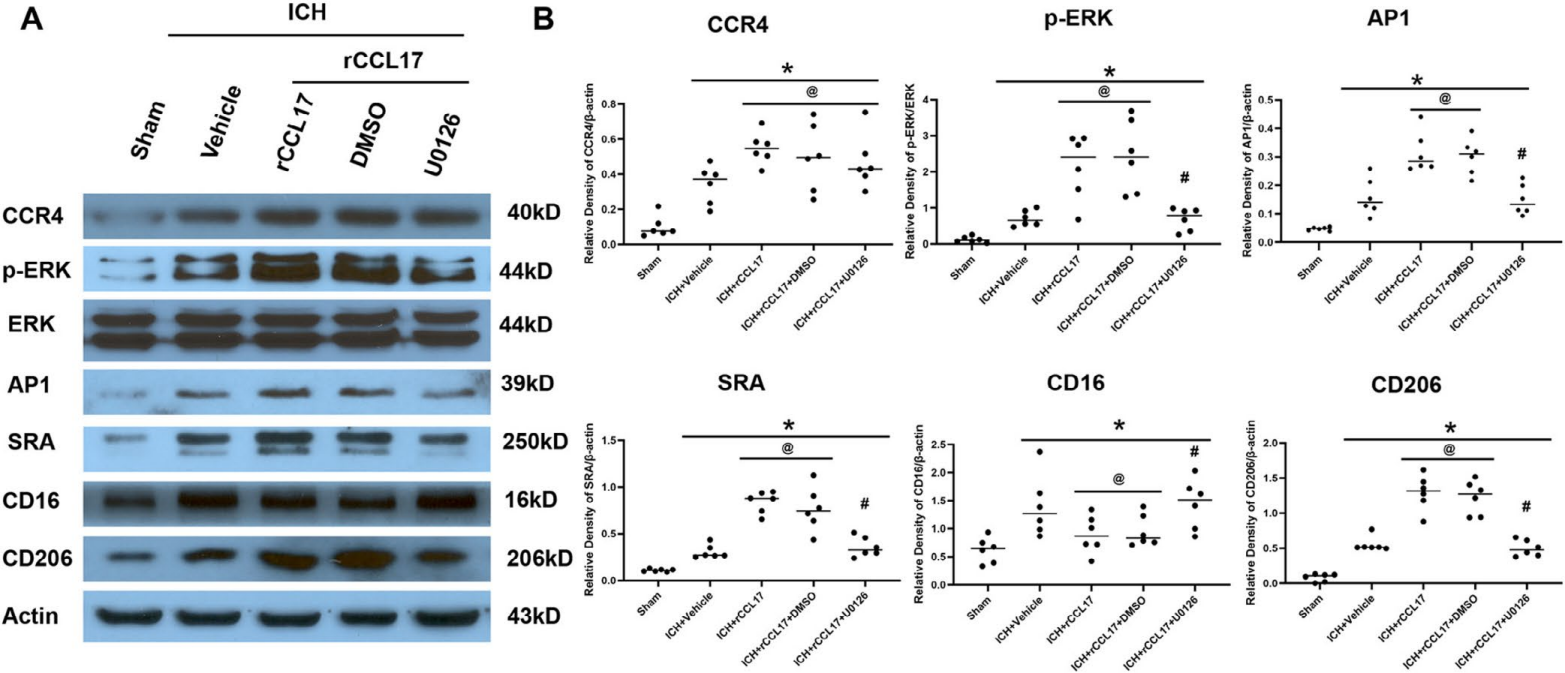
rCCL17 exerts the promotion of microglial phenotypic transition via ERK/AP‐1 signaling and SRA upregulation in the perihematomal region after ICH. (A) Western blot analysis of CCR4, p‐ERK, ERK, AP‐1, SRA, CD16, and CD206 expression in the ipsilateral hemisphere of sham, ICH + Vehicle, ICH + rCCL17, DMSO, and UD126 treated mice. (B) Quantitative analysis of the relative density of CCR4, p‐ERK, AP‐1, SRA, CD16, and CD206 protein expression. *n* = 6/group, *p < 0.05 versus sham, @ *p* < 0.05 versus ICH + Vehicle, # *p* < 0.05 versus ICH + rCCL17 + DMSO, Mean ± SD, One‐way ANOVA, Tukey test.

To elucidate the molecular mechanisms underlying the promotion of microglial phenotypic transition by rCCL17 in ICH, we examined its impact on the ERK/AP‐1 signaling pathway and the expression of the phagocytosis‐related receptor SRA in the perihematomal region. Western blot analysis of brain tissue lysates at 3 days post‐ICH revealed that rCCL17 treatment significantly increased ERK phosphorylation and AP‐1 expression compared to vehicle‐treated controls (Figure 8A,B). These findings indicate that rCCL17 activates the ERK/AP‐1 signaling pathway, which is involved in regulating neuronal survival and inflammatory responses.

To further explore the role of ERK signaling in rCCL17‐induced microglial phenotypic transition, we administered the ERK inhibitor U0126 in combination with rCCL17. U0126 treatment significantly reduced rCCL17‐induced ERK phosphorylation and AP‐1 expression (Figure 8A,B), suggesting that ERK activation is essential for rCCL17's downstream effects on AP‐1 signaling.

Importantly, rCCL17 treatment also significantly increased SRA expression on microglia in the perihematomal region compared to vehicle treatment (Figure 8A,B). This upregulation of SRA may enhance microglial phagocytosis, promoting the clearance of apoptotic neurons and debris, and supporting overall neuroprotection in the acute phase after ICH.

Interestingly, U0126 administration reduced rCCL17‐induced SRA upregulation (Figure 8A,B), suggesting that ERK activation plays a role in regulating SRA expression. These findings highlight the potential connection between ERK/AP‐1 signaling and the modulation of microglial phagocytosis by rCCL17.

## 4 Discussion

This study investigates the role of the CCL17/CCR4 axis in regulating microglial polarization and hematoma clearance following intracerebral hemorrhage (ICH), with a focus on the downstream ERK/AP1/SRA signaling pathway. Our findings highlight several key points [1]: CCR4 and the ERK/AP1/SRA signaling pathway are significantly upregulated in microglia in the perihematomal region after ICH [2]; CCR4 knockdown worsens neurological deficits, neuroinflammation, and hematoma volume, while recombinant CCL17 administration improves functional outcomes and provides neuroprotection [3]; the CCL17/CCR4 interaction activates the ERK/AP1/SRA signaling cascade, promoting a shift toward an anti‐inflammatory and phagocytic microglial phenotype [4]; inhibition of ERK impairs the beneficial effects of CCL17 on microglial polarization; and [5] the CCL17/CCR4 axis plays a central role in regulating microglial polarization and hematoma clearance through modulation of the ERK/AP1/SRA pathway. These results suggest that targeting this axis and its downstream signaling could offer a novel therapeutic strategy to enhance neurorepair and improve ICH outcomes.

CCR4's role in ICH‐induced brain injury is complex and multifaceted [30]. As a chemokine receptor, CCR4 is crucial for immune cell trafficking, particularly for Tregs and Th2 cells, which are key regulators in the brain–peripheral immune response [31, 32]. Our study demonstrates that CCR4 knockdown exacerbates outcomes, while CCR4 overexpression and CCL17 treatment provide neuroprotective effects. These findings align with the current understanding of chemokine‐mediated immune responses following CNS injury, where the interaction between the central nervous system and peripheral immune system plays a critical role in secondary brain injury [33]. Additionally, Bai et al. [34] highlighted the crucial role of peripheral blood immune cells in the immunological cascade following ischemic stroke, where monocytes influence outcomes by secreting cytokines or chemokines essential for monocyte recruitment, migration, and differentiation.

The elevated levels of endogenous CCL17 after ICH may originate from multiple sources through brain‐peripheral immune interactions. First, resident microglia, as the primary immune cells in the CNS, can be activated following ICH and produce CCL17. Second, peripheral macrophages that infiltrate the brain parenchyma through the compromised blood–brain barrier can secrete CCL17. Third, peripheral immune cells can produce CCL17 that enters the brain through systemic circulation, particularly when the blood–brain barrier is disrupted after ICH. These multiple sources of CCL17 reflect the complex interplay between central and peripheral immune responses following brain injury.

The protective effects of CCL17/CCR4 signaling may be attributed to several mechanisms. First, CCL17 can promote the infiltration of regulatory T cells (Tregs), which have been shown to suppress inflammatory responses and provide neuroprotection after hemorrhagic stroke. This is supported by recent evidence showing that increasing the number of Tregs in brain tissue leads to elevated levels of anti‐inflammatory cytokines such as IL‐10 and improved neurological outcomes. Second, CCR4 signaling may influence the phenotypic transformation of infiltrating immune cells, particularly monocytes/macrophages, promoting their conversion to an anti‐inflammatory M2 phenotype that facilitates tissue repair and recovery.

These findings are consistent with previous research demonstrating that exogenous CCL17 alleviates neuronal apoptosis and improves neurological function after subarachnoid hemorrhage [33]. Furthermore, recent studies have highlighted the crucial role of peripheral immune cells in the immunological cascade following stroke, where monocytes/macrophages influence outcomes through complex interactions with resident microglia and the secretion of various inflammatory mediators [35]. Understanding these intricate immune responses and their regulation through chemokine receptors like CCR4 may provide new therapeutic opportunities for treating ICH‐induced brain injury.

The CCL17/CCR4‐mediated shift in microglial polarization is a key mechanism underlying the neuroprotective effects of this signaling axis. Microglial cells, the resident immune cells of the central nervous system, can adopt different phenotypes in response to injury, particularly transitioning between pro‐inflammatory (M1) and anti‐inflammatory (M2) states [36, 37, 38]. Following ICH, microglia can polarize to M1, a pro‐inflammatory phenotype, or M2, a phenotype associated with tissue repair and inflammation resolution [39, 40]. Our results demonstrate that rCCL17 promotes M2 polarization and enhances the phagocytic activity of microglia/macrophages, facilitating recovery after ICH. The shift from M1 to M2 microglia correlates with improved functional outcomes, suggesting that promoting an anti‐inflammatory microglial phenotype is beneficial for tissue repair following ICH.

The molecular mechanisms underlying this shift involve the ERK/AP1/SRA signaling pathway. CCR4 activation by CCL17 triggers the ERK pathway [12], which mediates cellular responses including migration, proliferation, and survival of immune cells. The ERK pathway, part of the mitogen‐activated protein kinase (MAPK) cascade, is activated by various extracellular signals, including growth factors and cytokines [41, 42]. Upon activation, ERK translocates to the nucleus, where it influences the activity of transcription factors, including AP‐1. AP‐1 is a multifunctional transcription factor that plays a critical role in regulating gene expression related to cell proliferation, survival, inflammation, and cancer [43]. It influences the expression of SRA, a receptor essential for phagocytosis and the clearance of cellular debris [44].

AP1, a downstream transcription factor, regulates the expression of pro‐inflammatory cytokines and chemokines, influencing the immune response and SRA expression. Our study shows that rCCL17 activates this pathway, driving microglial polarization toward an anti‐inflammatory and phagocytic phenotype. Moreover, ERK inhibition diminishes the beneficial effects of CCL17 on microglial transition, confirming the importance of this signaling cascade in promoting the M2 phenotype. SRA plays a crucial role in phagocytosis, helping clear cellular debris and pathogens [44]. It is highly expressed on macrophages and dendritic cells and is essential for maintaining tissue homeostasis and modulating inflammation [45]. The multifunctional nature of SRA makes it vital for regulating inflammation, maintaining tissue homeostasis, and contributing to the immune response [46]. Our study shows that rCCL17 enhances SRA expression on microglia, supporting the transition toward the M2 phenotype and facilitating hematoma clearance. This suggests that the neuroprotective effects of CCL17 are, in part, mediated by SRA expression, which promotes phagocytosis and inhibits M1 microglial polarization.

While our study provides valuable insights, there are several limitations. First, we used an adult male mouse model of autologous blood injection ICH and focused on the acute phase, without evaluating long‐term effects. Future studies should investigate the protective effects of CCL17 in different age groups, sexes, and alternative ICH models, such as the collagenase‐induced model. Second, we did not assess protein expression at the gene level, which could provide further mechanistic insights. Third, given that AP1 and SRA on microglia suppress inflammatory responses, it is possible that rCCL17 enhances hematoma clearance through their anti‐inflammatory actions. Future studies should use various inhibitors of the CCR4/ERK/AP1/SRA signaling pathway to explore their role in hematoma clearance and functional recovery. Lastly, the role of CCL17 on T cells and the mechanisms by which Tregs influence microglial polarization from M1 to M2 are not addressed here and warrant further exploration.

## 5 Conclusion

In conclusion, our findings suggest that the CCL17/CCR4 axis plays a pivotal role in microglial polarization and hematoma clearance after ICH, with potential therapeutic implications. Targeting this pathway could enhance neurorepair mechanisms and improve outcomes for ICH patients, but further research is needed to validate these findings in human models and explore clinical applications.

## Author Contributions

Shuixiang Deng and Yao Wang participated in the experimental design. Shuixiang Deng and Peng Jin conducted the experiments. Yao Wang analyzed the data, and Shuixiang Deng revised the paper. Xianglan Chen and Junjie Jin participated in the experimental design and manuscript preparation. All authors read and approved the final manuscript.

## Conflicts of Interest

The authors declare no conflicts of interest.

## Supporting information


**Figure S1.** Experimental Flowchart for Studying CCL17/CCR4‐Mediated Microglial Polarization After Intracerebral Hemorrhage This flowchart outlines the experimental design used to investigate the role of the CCL17/CCR4 axis in microglial polarization via the ERK/AP1/SRA pathway after intracerebral hemorrhage. The diagram details the step‐by‐step experimental procedures, including the induction of hemorrhage, treatment interventions, time points for sample collection, and the analytical methods employed to assess the effects on microglial behavior and hematoma resolution.

**Figure S2.** A flowchart illustrating the key steps in the activation of microglia via the CCL17/CCR4 signaling pathway following intracerebral hemorrhage (ICH). Resting microglia are activated by rCCL17, which binds to the CCR4 receptor, initiating the phosphorylation of ERK. This leads to the activation of the AP‐1 transcription factor and subsequent polarization of microglia into M1 and M2 phenotypes. The M1 microglia promote the enlargement of the hematoma, while M2 microglia facilitate hematoma removal through their reparative function.

## Data Availability

The data that support the findings of this study are available on request from the corresponding author. The data are not publicly available due to privacy or ethical restrictions.
