# Supplementary figures and images for: CCL17/CCR4 Axis Promotes Hematoma Clearance via ERK/AP1/SRA‐Mediated Microglial Polarization After Intracerebral Hemorrhage
Source: CNS Neurosci Ther. 2025 Feb 25;31(2):e70288. doi: 10.1111/cns.70288 (PMC11851156; doi:10.1111/cns.70288)

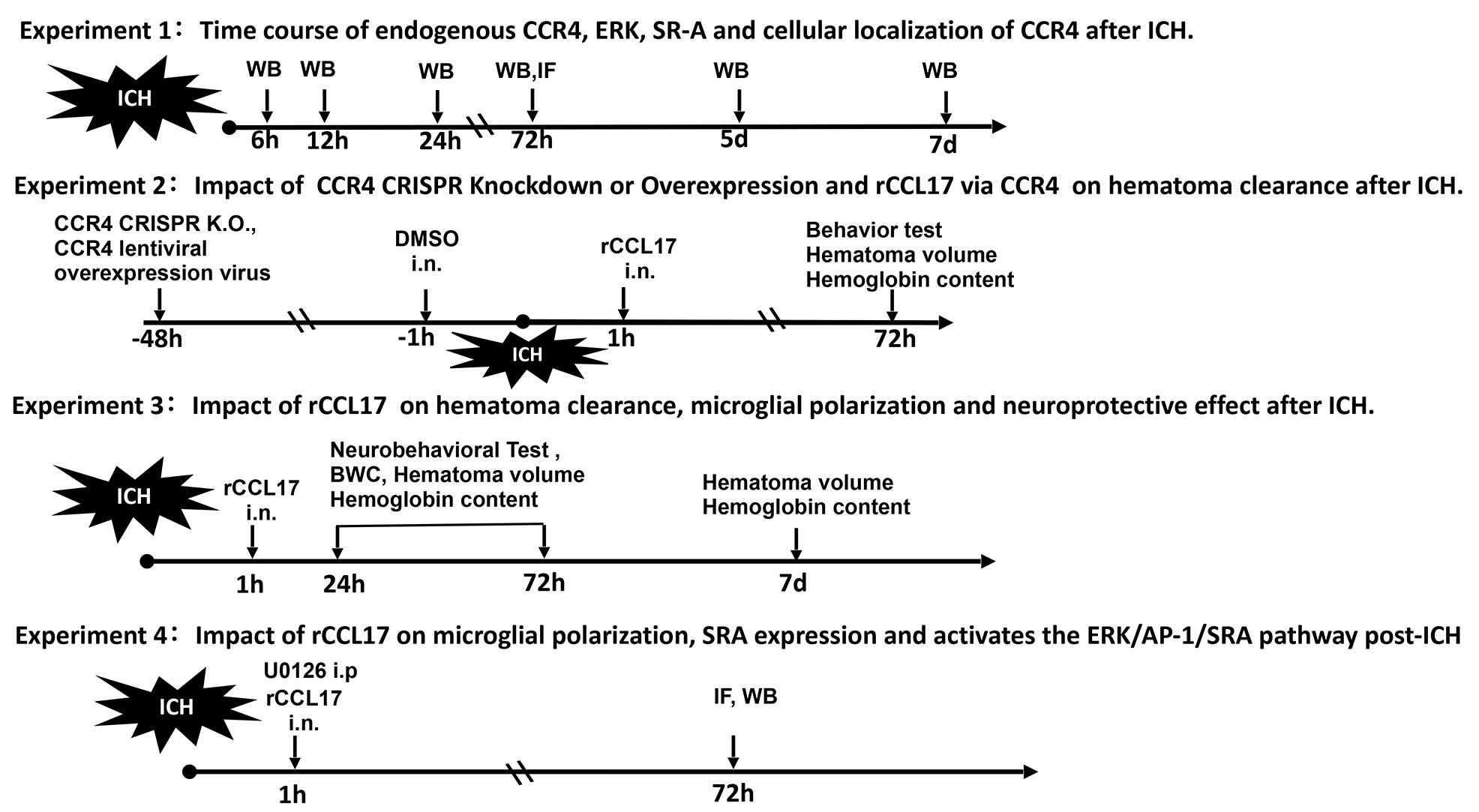

Supplement: Supplementary file 1 — Figure S1. Experimental Flowchart for Studying CCL17/CCR4‐Mediated Microglial Polarization After Intracerebral Hemorrhage This flowchart outlines the experimental design used to investigate the role of the CCL17/CCR4 axis in microglial polarization via the ERK/AP1/SRA pathway after intracerebral hemorrhage. The diagram details the step‐by‐step experimental procedures, including the induction of hemorrhage, treatment interventions, time points for sample collection, and the analytical methods employed to assess the effects on microglial behavior and hematoma resolution. [file CNS-31-e70288-s002.tif]

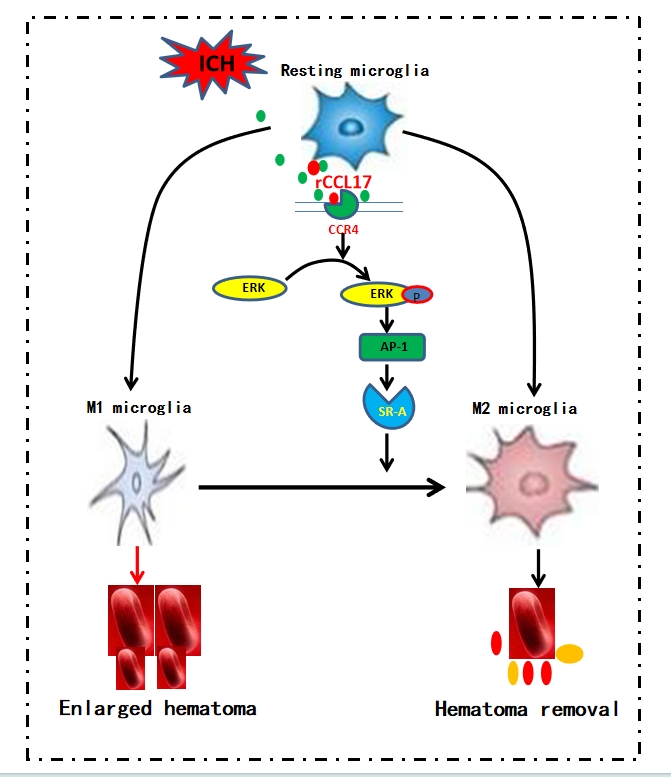

Supplement: Supplementary file 2 — Figure S2. A flowchart illustrating the key steps in the activation of microglia via the CCL17/CCR4 signaling pathway following intracerebral hemorrhage (ICH). Resting microglia are activated by rCCL17, which binds to the CCR4 receptor, initiating the phosphorylation of ERK. This leads to the activation of the AP‐1 transcription factor and subsequent polarization of microglia into M1 and M2 phenotypes. The M1 microglia promote the enlargement of the hematoma, while M2 microglia facilitate hematoma removal through their reparative function. [file CNS-31-e70288-s001.jpg]
